# Salud Mesoamérica 2015 Initiative: design, implementation, and baseline findings

**DOI:** 10.1186/s12963-015-0034-4

**Published:** 2015-02-07

**Authors:** Ali H Mokdad, Katherine Ellicott Colson, Paola Zúñiga-Brenes, Diego Ríos-Zertuche, Erin B Palmisano, Eyleen Alfaro-Porras, Brent W Anderson, Marco Borgo, Sima Desai, Marielle C Gagnier, Catherine W Gillespie, Sandra L Giron, Annie Haakenstad, Sonia López Romero, Julio Mateus, Abigail McKay, Ali A Mokdad, Tasha Murphy, Paria Naghavi, Jennifer Nelson, Miguel Orozco, Dharani Ranganathan, Benito Salvatierra, Alexandra Schaefer, Gulnoza Usmanova, Alejandro Varela, Shelley Wilson, Sarah Wulf, Bernardo Hernandez, Rafael Lozano, Emma Iriarte, Ferdinando Regalia

**Affiliations:** Institute for Health Metrics and Evaluation, 2301 5th Ave, Suite 600, Seattle, WA USA; Salud Mesoamérica 2015/Inter-American Development Bank, Calle 50, Edificio Tower Financial Center (Towerbank), Piso 23, Panamá, Panamá; Universidad de Costa Rica, Ciudad Universitaria Rodrigo Facio Brenes, San Jose, Costa Rica; RAND, 1776 Main Street, Santa Monica, CA 90401 USA; UNIMER, Calle Los Abetos Pje. 4 N° 3 Col. San Francisco, San Salvador, El Salvador; Children’s National Health System, 111 Michigan Avenue NW, Washington DC, USA; FES Foundation, Avenida 8 Norte 22AN-15, Colombia, Sur América; University of Belize, Belmopan, Belize; Department of Surgery, University of Texas Southwestern, Dallas, TX USA; University of Washington School of Social Work, 4101 15th Avenue N, Seattle, WA USA; Centro de Investigacion y Estudios de la Salud de la Universidad Nacional Autónoma de Nicaragua (CIES-UNAN), Managua, Nicaragua; George Washington University, 950 New Hampshire Ave, NW, Washington DC, USA; Health Departament, El Colegio de la Frontera Sur, Carretera panamericana y periférico sur s/n, Barrio Maria Auxiliadora, CP 20290 San Cristóbal de las Casas, Chiapas Mexico; Inter-American Development Bank, 1300 New York Avenue, NW, Washington, D.C, USA

**Keywords:** Results-based financing, Salud Mesoamerica 2015, Vaccination, Contraceptives, Skilled birth attendance, Antenatal care, Anemia, Wasting, Health facilities

## Abstract

**Background:**

Health has improved markedly in Mesoamerica, the region consisting of southern Mexico and Central America, over the past decade. Despite this progress, there remain substantial inequalities in health outcomes, access, and quality of medical care between and within countries. Poor, indigenous, and rural populations have considerably worse health indicators than national or regional averages. In an effort to address these health inequalities, the Salud Mesoamérica 2015 Initiative (SM2015), a results-based financing initiative, was established.

**Methods:**

For each of the eight participating countries, health targets were set to measure the progress of improvements in maternal and child health produced by the Initiative. To establish a baseline, we conducted censuses of 90,000 households, completed 20,225 household interviews, and surveyed 479 health facilities in the poorest areas of Mesoamerica. Pairing health facility and household surveys allows us to link barriers to care and health outcomes with health system infrastructure components and quality of health services.

**Results:**

Indicators varied significantly within and between countries. Anemia was most prevalent in Panama and least prevalent in Honduras. Anemia varied by age, with the highest levels observed among children aged 0 to 11 months in all settings. Belize had the highest proportion of institutional deliveries (99%), while Guatemala had the lowest (24%). The proportion of women with four antenatal care visits with a skilled attendant was highest in El Salvador (90%) and the lowest in Guatemala (20%). Availability of contraceptives also varied. The availability of condoms ranged from 83% in Nicaragua to 97% in Honduras. Oral contraceptive pills and injectable contraceptives were available in just 75% of facilities in Panama. IUDs were observed in only 21.5% of facilities surveyed in El Salvador.

**Conclusions:**

These data provide a baseline of much-needed information for evidence-based action on health throughout Mesoamerica. Our baseline estimates reflect large disparities in health indicators within and between countries and will facilitate the evaluation of interventions and investments deployed in the region over the next three to five years. SM2015’s innovative monitoring and evaluation framework will allow health officials with limited resources to identify and target areas of greatest need.

**Electronic supplementary material:**

The online version of this article (doi:10.1186/s12963-015-0034-4) contains supplementary material, which is available to authorized users.

## Background

In the past decade, population health has improved markedly in Mesoamerica, the region consisting of southern Mexico and Central America [1-3]. Despite this progress, substantial inequalities in health outcomes, access, and quality of medical care remain between and within countries [4,5]. Vulnerable groups including poor, indigenous, and rural populations have considerably worse health indicators than national or regional averages [6-16].

The Salud Mesoamérica 2015 Initiative (SM2015) was launched to address these health inequalities in eight countries: El Salvador, Guatemala, Honduras, Nicaragua, Belize, Costa Rica, Panama, and Mexico. Administered by the Inter-American Development Bank (IDB), the Initiative is a public-private partnership of the Bill & Melinda Gates Foundation, the Carlos Slim Health Institute, Spain’s Cooperation Agency for International Development, and the ministries of health in these Mesoamerican countries. SM2015 harnesses a results-based financing approach to deliver integrated, evidenced-based supply- and demand-side interventions. Deploying incentives to increase the use and quality of health services for the poorest quintile of the population is a major aspect of this approach.

In cooperation with governments, the Initiative established a core set of goals focused on maternal and child health for the poorest 20% of the population in each country. Meeting Millennium Development Goals 4 and 5 in these areas is a top objective for the Initiative. SM2015 also aims to reduce chronic malnutrition, decrease anemia in children, improve completion of vaccination schedules, and increase the number of births attended by skilled personnel.

In this manuscript, we describe the design, implementation, and baseline findings of the SM2015 evaluation conducted by the Institute for Health Metrics and Evaluation (IHME) in collaboration with IDB. These results are sourced from several of the largest, comparable cross-country household and health facility surveys in the region. These findings, and their comparison with follow-up measurements forthcoming, will inform the investment decisions during the rollout of the Initiative.

## Methods

As a results-based financing mechanism, the sequence of SM2015 funding, interventions, and evaluation components is interdependent and deeply integral to the successful implementation of the Initiative. At the onset of SM2015, an initial contribution (investment tranche), accompanied by counterpart financing from the government in each country, financed preliminary child and maternal health interventions. Interventions included the implementation of the Essential Obstetric and Neonatal Care (EONC) strategy, strengthening of referral networks, improving the supply chain, encouraging the adaptation of services for indigenous populations, supporting new service delivery platforms and community platforms, and the design and approval of updated country norms and protocols, among other activities. Follow-up measurements at 18, 36, and 54 months will capture the impact of the interventions. Depending on whether targeted improvements are met at each of these critical junctures, countries will be reimbursed with funds corresponding to half of the counterpart investment to be used freely within the health sector.

The set of performance indicators and targets were set with governments, in line with country-specific priorities in maternal and child health. Key indicators include coverage of contraceptives, antenatal and postnatal care for women and newborns, in-facility delivery and skilled birth attendance, management of maternal and neonatal complications, complete vaccination coverage for age, prevalence of anemia in children, and quality of care for antenatal, delivery, postnatal, and child health care visits. Indicator targets were set based on literature reviews of intervention effectiveness from previous country-level studies, trend analysis using data from the Global Burden of Disease 2010 Study [2,17-21], expert advice, and a cost-benefit model developed by IDB. Additional file 1: Table S1 presents these indicators for each country.

We designed surveys specifically tailored to each indicator and country context. Surveys were conducted in both households and health facilities in order to assess coverage of health services, barriers to care, and population health outcomes, alongside health system infrastructure and service delivery components. Specific to Costa Rica, school-based questionnaires were administered in order to assess indicators related to sexual and reproductive health and the prevention of pregnancy among teenagers. Surveys were conducted in both intervention and control areas in Honduras, Nicaragua, Guatemala, and Mexico. Households were asked to indicate which health facilities were visited for different types of care, allowing us to link household experience and outcomes with facility conditions and services.

For the household survey, we included all SM2015 municipalities in our sampling frame, stratified by intervention and control (where applicable), and selected a random sample from the list of all localities, using probability proportional to size. These localities contained approximately 150 households and were the primary sampling unit (PSU). We did not stratify by poor, indigenous, or rural populations, as our sampling design ensured the inclusion of PSUs from all the SM2015 areas depending on their size. We then conducted our own census in each selected PSU. These censuses accounted for the movement of poor populations in and out of study areas in the time since the most recent national census and provided the most accurate and up-to-date housing and population data for our sampling frames. Using results from the census, we randomly selected 30 eligible households (with women aged 15 to 49 years or children under 5 years) in each PSU.

The household survey consisted of three components: 1) Household Characteristics Questionnaire, 2) Maternal and Child Questionnaire, and 3) Physical Measurements Module. The Household Characteristics Questionnaire collected information on socio-economic factors, assets, expenditure, and health expenses. Moreover, we collected information on the source of water, type of toilet facilities, exposure to secondhand smoke, ownership of various assets (durable goods, land, livestock, etc.), household expenses, and sources of health care financing. The Maternal and Child Questionnaire collected information from all women of reproductive age (15 to 49 years) in the household. Women were asked questions on the following topics: background characteristics (including education, occupation, and exposure to media), access to health care, current health status, recent history of illness and associated medical expenses, complete birth history, fertility preferences, knowledge and use of family planning methods (including barriers to use), exposure to health system interventions, and satisfaction with community health workers. Women with children aged 0 to 5 years were asked detailed questions in reference to each child born in the past five years on topics such as: birth spacing, antenatal care, labor and delivery, postpartum care, breastfeeding and infant feeding practices, child’s current health status, child’s recent history of illness including diarrhea, fever, and acute upper respiratory infection and associated medical expenses, child’s exposure to health system interventions, immunization, and supplementation history.

For the Physical Measurements Module, medically trained personnel performed physical assessments, capturing weight, height/length, and hemoglobin levels of children aged 0 to 59 months. Portable scales and stadiometers were used for the anthropometric measurements. Height and weight measurements were used to assess prevalence of wasting, stunting, underweight, and overweight in young children. Hemoglobin levels were assessed in the field using a portable HemoCue™ machine. In Mexico and Nicaragua, samples of capillary blood were collected from children 12 to 23 months using the dried blood spot (DBS) technique to measure the presence of measles antibodies and assess effective coverage of measles immunization. DBS samples were shipped to laboratories at the National Institute of Public Health of Mexico for analysis. Additionally, in Panama, water quality tests were performed in three randomly-selected households within each PSU to assess chlorine concentrations and the presence of coliforms.

The health facility survey collected data on facility conditions, service provision and utilization, and quality of care. The survey involved three main components: an interview questionnaire, an observation checklist, and medical record reviews (MRRs). Health facilities were grouped according to three levels of EONC – ambulatory, basic, and complete – as provided by SM2015. Different criteria were assessed depending on the EONC classification level. In the interview questionnaire, the facility director, manager, or other person in charge of the health facility was interviewed to capture information on general facility characteristics, infrastructure, human resource composition, supply logistics, infection control, child health care, vaccine availability, family planning service provision, availability of contraceptives, and antenatal, delivery, and postpartum care. Once completed, surveyors used an observation checklist to record direct observations of the availability and functionality, as applicable, of essential equipment and supplies required for maternal and child health care, including pharmaceuticals. Surveyors also reviewed administrative records of pharmaceutical stocks in this module, capturing drug stock-outs occurring in the three months prior to the survey. We used MRRs to capture retrospective data on record-keeping and treatment practices of surveyed facilities. The MRRs covered various medical complications that mothers and infants experienced during delivery and how each case was treated at a given health facility. The MRRs also captured the medical practices of the facilities before, during, and after uncomplicated births. Depending on the country, other MRRs on diarrhea, pneumonia, low birth weight, child registration, deworming, and family planning services offered were also implemented.

The SM2015 surveys were conducted using a computer‐assisted personal interview (CAPI). CAPI was programmed using DatStat Illume and installed on netbooks, which allowed surveyors to input data in real time throughout survey implementation. The use of CAPI also permitted instantaneous data transfer via a secure EMBED to IHME when surveyors were connected to the internet. IHME led training sessions and pilots in each country before implementation. Surveys were conducted in Spanish and other indigenous languages when applicable. During data collection, data were continuously monitored by IHME for quality assurance. The study received institutional review board (IRB) approval from the University of Washington, partnering data collection agencies, and the Ministry of Health in each country. Analyses were conducted using Stata versions 12.1 and 13.1.

### Mexico

In the state of Chiapas, 30 intervention and 26 control municipalities with similar socio-economic characteristics and ethnic composition were designated for the study. These municipalities were divided into 8,163 segments and a representative sample of 181 segments was selected with probability proportional to size, where size was represented by the number of occupied households within the segment, as captured in the 2010 Mexico Population Census. A sample of 90 health facilities (60 intervention and 30 control facilities) was selected from a list of all facilities serving the 56 municipalities. The final sample included 12 facilities that offer complete EONC, 18 facilities that offer basic EONC, and 60 facilities that offer ambulatory EONC. For the medical record review, a systematic sampling method was used to reach the required sample of complications and delivery records in each facility, with some records for some types of complications oversampled. Cases of maternal and neonatal complications were sampled at random from Ministry of Health (Instituto de Salud del Estado de Chiapas) registries.

The baseline survey was carried out between July 25, 2012 and May 18, 2013. In total, 24,349 households were identified in our census and 5,428 households were interviewed (3,877 intervention and 1,551 control households). The response rate was 99% for the SM2015 Household Census and 97% for the Household Characteristics Questionnaire. Using information gathered from the household roster, women of reproductive age were identified from the subsample of interviewed households as eligible for the Maternal and Child Questionnaire. Of these, 6,988 successfully completed the questionnaire, yielding a 95% response rate. The household roster was also used to identify children aged 0 to 59 months as eligible for the Physical Measurements Module among the interviewed households. In total, 6,499 of these children were measured, yielding a 99% response rate.

### Honduras

In Honduras, 19 intervention municipalities were identified on the basis of their high concentration of the country’s lowest wealth quintile. An additional 16 control municipalities with similar socio-economic characteristics and ethnic composition were identified as well. These municipalities were divided into 3,021 segments, and a representative sample of 99 segments was selected using probability proportional to size, where size was represented by the number of occupied households within the segment, as captured in the 2011 National Health Survey (ENDESA). A sample of 90 health facilities (60 intervention and 30 control facilities) was selected from a list of all facilities serving the 35 municipalities. Of the original sample, one control facility split in two during the time between the generation of the list and the interview, and thus was surveyed as two separate facilities. Four facilities in intervention areas could not be interviewed: one did not give consent and three others were inaccessible due to security reasons. Of those, three facilities were replaced with randomly selected ambulatory facilities within the same municipality, or if no more facilities were present in that municipality, a neighboring one. The final sample included 59 facilities in intervention areas and 31 facilities in control areas. For the MRR, a systematic sampling method was used to reach the required sample of records in each facility. Records for specific conditions (maternal and neonatal complications, deliveries, antenatal and postpartum care, and child care) were selected according to a quota set considering the EONC level of each facility. Cases of maternal and neonatal complications were sampled at random from Ministry of Health (Secretaría de Salud) registries.

The baseline survey was carried out between January 17, 2013 and June 1, 2013. In total, 15,741 households were identified in our census, and 2,971 households were interviewed (1,526 intervention and 1,445 control). The response rate was 99.9% for the SM2015 Household Census and 99% for the Household Characteristics Questionnaire. A total of 3,580 women of reproductive age successfully completed the Maternal and Child Health Questionnaire, yielding a response rate of 86%. Among eligible children aged 0 to 59 months, 3,192 children were measured for the Physical Measurements Module, yielding a 97% response rate.

### Nicaragua

SM2015 was implemented in municipalities belonging to three local health systems, or SILAIS (Jinotega, Matagalpa, and the North Atlantic Region), and with the highest rates of unsatisfied basic needs. In Nicaragua, 19 intervention municipalities and four control municipalities with similar socio-economic characteristics and ethnic composition were identified. We divided these municipalities into 1,455 segments, and 90 were selected using probability proportional to size, where size was represented by the number of occupied households within the segment, as captured on the 2005 Nicaragua Population Census. A sample of 90 (60 intervention and 30 control) health facilities was selected from a list of all facilities serving the 23 municipalities. Data collection in Nicaragua faced a number of challenges particularly related to security. Due to these safety problems, specifically in the North Atlantic Region, data collection had to be stopped. Therefore, only 40 facilities in intervention areas and 24 facilities in control areas were surveyed. To make sure that no bias was introduced, we used data from the most recent national census to compare the characteristics of surveyed and non-surveyed areas. We found no major differences with respect to general household characteristics, poverty index, age, average distance to the nearest health facility, and coverage of antenatal care and institutional delivery.

The baseline survey was carried out between March 1, 2013 and August 29, 2013. In total, 8,867 households were identified in our census, and 2,071 households were interviewed (1,300 intervention and 771 control). The response rate was nearly 100% for the SM2015 Household Census and 94% for the Household Characteristics Questionnaire. A total of 2,823 women of reproductive age successfully completed the Maternal and Child Health Questionnaire, yielding a response rate of 92%. Among eligible children aged 0 to 59 months, 2,236 children were measured for the Physical Measurements Module, yielding a 99% response rate.

### Guatemala

SM2015 was carried out in intervention municipalities from two departments (San Marcos and Huehuetenango) on the basis of their high concentration of residents in the country’s lowest wealth quintile. There were 17 intervention municipalities and 10 control municipalities with similar socio-economic characteristics and ethnic composition. The 27 municipalities were divided into 1,033 segments, and a sample of 148 segments was selected using probability proportional to size, where size was represented by the number of occupied households within the segment, as captured in the 2002 Guatemala Population Census. A sample of 93 (64 interventions and 29 control) health facilities was selected from a list of all facilities serving the 27 municipalities.

The baseline survey was carried out between April 15, 2013 and August 11, 2013. In total, 20,451 households were identified in our census and 4,420 households were interviewed (3,546 intervention and 874 controls). The response rate was nearly 100% for the SM2015 Household Census and 93% for the Household Characteristics Questionnaire. A total of 5,899 women of reproductive age successfully completed the Maternal and Child Health Questionnaire, yielding a response rate of 90%. Among eligible children aged 0 to 59 months, 5,404 children were measured for the Physical Measurements Module, yielding a 93% response rate.

### El Salvador

SM2015 was implemented in 14 municipalities on the basis of their high concentration of residents in the country’s lowest wealth quintile. The 14 targeted municipalities were divided into 523 segments, and a sample of 139 segments was selected using probability proportional to size, where size was represented by the number of occupied households within the segment, as captured in the 2007 El Salvador Census. A sample was drawn randomly from a list of all facilities that provide health services to the 139 segments in intervention areas. In total, 55 basic health units (ECOS) and 10 specialized health units (three specialized ECOS and seven health centers) were included in our sample.

The baseline survey was carried out between March 1, 2011 and July 8, 2011. In total, 16,178 households were identified in our census, and 3,625 households were interviewed in intervention areas. The response rate was 88.0% for the SM2015 Household Census and 92.1% for the Household Characteristics Questionnaire. A total of 4,730 women of reproductive age successfully completed the Maternal and Child Health Questionnaire, yielding a response rate of 90.6%. Among eligible children aged 0 to 59 months, 3,328 children were measured for the Physical Measurements Module, yielding an 86.8% response rate.

### Panama

SM2015 was implemented in Kuna Yala and Emberá in Panama based on the high concentration of residents in the country’s lowest wealth quintile. These areas were divided into 158 segments, and a sample of 61 segments was selected using probability proportional to size, where size was represented by the number of occupied households within the segment, as captured in the 2010 Panama Population Census. All functioning Ministry of Health facilities offering ambulatory and basic EONC in the area were included, a total of 38 facilities. In three households in each segment, we conducted water quality tests. Trained data collectors took samples of the household’s drinking water source. These samples were tested for the concentration of chlorine and for the presence of coliforms.

The baseline survey was carried out between April 2, 2013 and August 31, 2013. In total, 4,947 households were identified in our census, and 1,710 households were in intervention areas. The response rate was nearly 100% for the SM2015 Household Census and 95% for the Household Characteristics Questionnaire. A total of 2,453 women of reproductive age completed the Maternal and Child Health Questionnaire, yielding a response rate of 82%. Among eligible children aged 0 to 59 months, 2,253 children were measured for the Physical Measurements Module, yielding a 93% response rate.

### Belize

SM2015 was implemented in three districts (Corozal, Orange Walk, and Cayo) based on the high concentration of residents in the country’s lowest wealth quintile. The baseline survey was carried out between April 18, 2013 and May 3, 2013. Because funds were limited, only $750,000 was allotted to SM2015 in Belize, and a community survey was implemented in lieu of a household survey to collect information on 350 families in Belize. For efficiency, we chose to interview 175 families approached in markets and town centers and 175 families in their homes. Due to the nature of the convenience sampling survey (not requiring probabilistic sampling) for the community survey, response rates cannot be calculated.

All facilities serving these communities were identified using a referral network outlined by the Ministry of Health. The sampling frame contained 40 facilities, representing all three levels of EONC: ambulatory, basic, and complete. All facilities were sampled. However, one facility was found to be nonfunctioning and thus, in total, 39 facilities were surveyed.

### Costa Rica

In Costa Rica, SM2015 focuses on adolescents and was implemented in the 11 health areas that encompass the poorest districts in the country. We implemented a school-based survey in the selected area. A random sample of 39 schools was selected from a total of 150 schools in the three areas. In each selected school, one class of each grade was selected at random to be included in the study. All students in the selected groups were invited to participate in the study. The school-based survey consisted of a paper-based questionnaire completed by students in the classroom. The questionnaire captured knowledge, attitudes, and behaviors related to sexual and reproductive health, as well as contact with reproductive health services among respondents in grades 7 through 11.

In total, 3,239 students were selected for the study, and 924 students completed the survey, yielding a response rate of 28.5%. While this response rate was lower than desired, it is not surprising given the topic of the survey, the level of engagement of families in the region surveyed, and the response rates observed in similar surveys [22,23].

## Results

**Figure 1:**
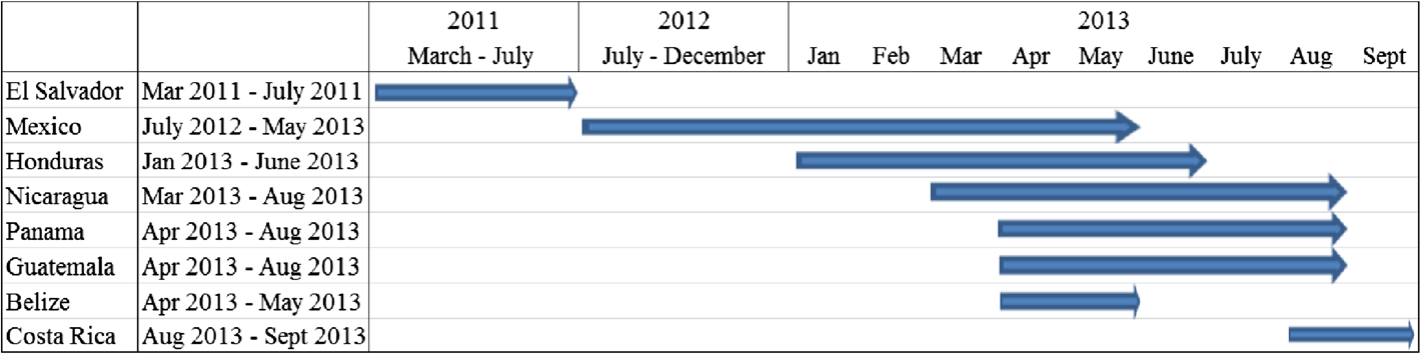
**Data collection timeline.**

Across all eight countries, we conducted censuses capturing over 90,000 occupied households, completed 20,225 household interviews, and surveyed 479 health facilities. Baseline data collection began in El Salvador in March of 2011 and ended with Costa Rica in September of 2013. The timeline of data collection by country is shown in Figure 1, depicting the concentration of data collection in April and May of 2013. Table 1 summarizes the samples in each country, disaggregated by intervention and control areas. Table 2 summarizes the health facility sample disaggregated by the EONC services that each unit provides.

**Table 1:** **Sample description by country**

**Country**	Census*		**Household**		**Women**		**Children**		**Health facilities**		**Students**
	***Intervention***	***Control***	***Intervention***	***Control***	***Intervention***	***Control***	***Intervention***	***Control***	***Intervention***	***Control***	***Intervention***
Belize**					351		311		39		
Costa Rica***											924
El Salvador	16,178		3,625		4,730		3,328		65		
Guatemala	16,847	3,604	3,546	874	4,658	1,241	4,224	1,058	64	29	
Honduras	8,132	7,609	1,526	1,445	1,868	1,474	1,622	1,522	59	31	
Mexico	17,471	6,878	3,877	1,551	5,016	1, 972	4,635	1,827	60	30	
Nicaragua	5,698	3,169	1,300	771	1,720	1,103	1,407	818	40	24	
Panama	4,947		1,710		2,453		2,253		38		
Total	69273	21260	15584	4641	20796	3818	17780	5225	365	114	924

*Reflects total number of occupied households counted by the SM2015 Census (90,533). Of these, 88,546 completed the census questionnaire.

**Convenience community survey.

***Surveys conducted in 41 schools capturing responses from 365 boys and 555 girls in grades 7 to 11.

**Table 2:** **Health facility sample by EONC classification**

**Country**	**Ambulatory EONC**		**Basic EONC**		**Complete EONC**	
	***Intervention***	***Control***	***Intervention***	***Control***	***Intervention***	***Control***
Belize	35		2		2	
El Salvador	58		7			
Guatemala	47	21	13	7	4	1
Honduras	45	20	8	7	6	4
Mexico	41	19	11	7	8	4
Nicaragua	32	23	5	1	3	
Panama	21		17			
Total	279	83	63	22	23	9

EONC: essential obstetric and neonatal care.

The distribution of weight-for-height z-scores according to the 2006 World Health Organization growth standards [24] in all countries is shown in Table 3. Wasting, defined as a z-score of less than or equal to −2 was 1% in all countries except Panama and El Salvador (2% and 3%, respectively). The proportion of overweight children, defined as a z-score of greater than or equal to 2, varied by country, with the highest levels seen in Nicaragua (8%) and the lowest observed in Panama (3%).

**Table 3:** **Distribution of weight by height z-score by country**

**Weight-for-height z-score**	**Guatemala (N = 4723)**		**Honduras (N = 2875)**		**Mexico (N = 5755)**	
	**%**	**SE**	**%**	**SE**	**%**	**SE**
less than −3	0%	0%	0%	0%	0%	0%
−3 to −2	1%	0%	1%	0%	1%	0%
−2 to 0	39%	1%	40%	1%	30%	1%
0 to 2	54%	1%	54%	1%	62%	1%
2 to 3	3%	0%	4%	0%	5%	0%
greater than 3	2%	0%	2%	0%	2%	0%
**Weight-for-height z-score**	**Nicaragua (N = 2158)**		**Panama (N = 1847)**		**El Salvador (N = 3273)**	
	**%**	**SE**	**%**	**SE**	**%**	**SE**
less than −3	0%	0%	1%	0%	1%	0%
−3 to −2	1%	0%	1%	0%	2%	0%
−2 to 0	35%	1%	42%	2%	41%	1%
0 to 2	57%	1%	53%	2%	51%	1%
2 to 3	5%	1%	2%	0%	4%	0%
greater than 3	3%	1%	1%	0%	2%	0%

**Figure 2:**
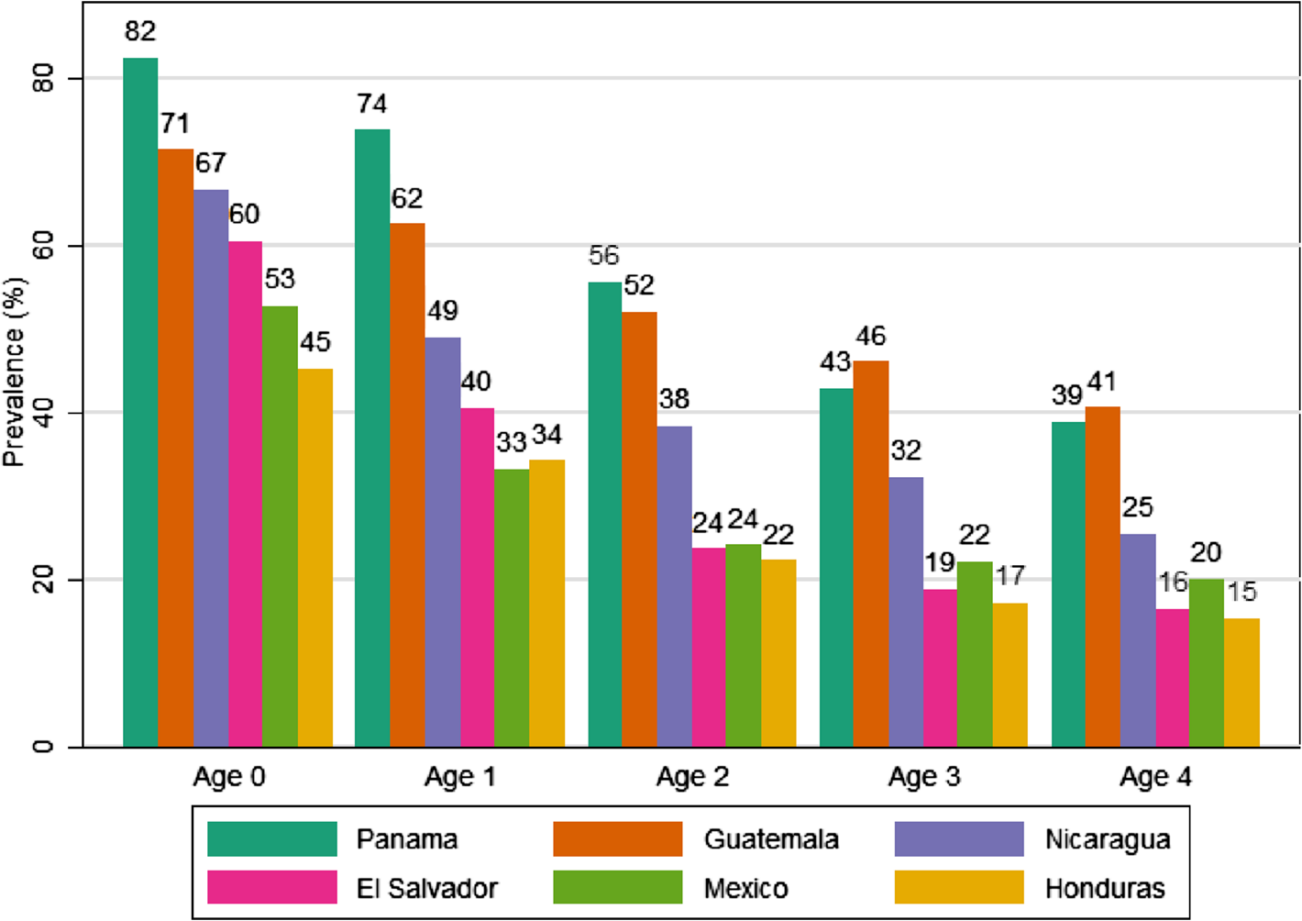
**Anemia prevalence by age and country.**

Figure 2 shows the distribution of anemia by age in each country, based on hemoglobin measurements. Overall, anemia declined with age and, across countries, the highest levels were consistently observed among children aged 0 to 11 months. Anemia was most prevalent in Panama and Guatemala and least prevalent in Honduras and Mexico.

**Figure 3:**
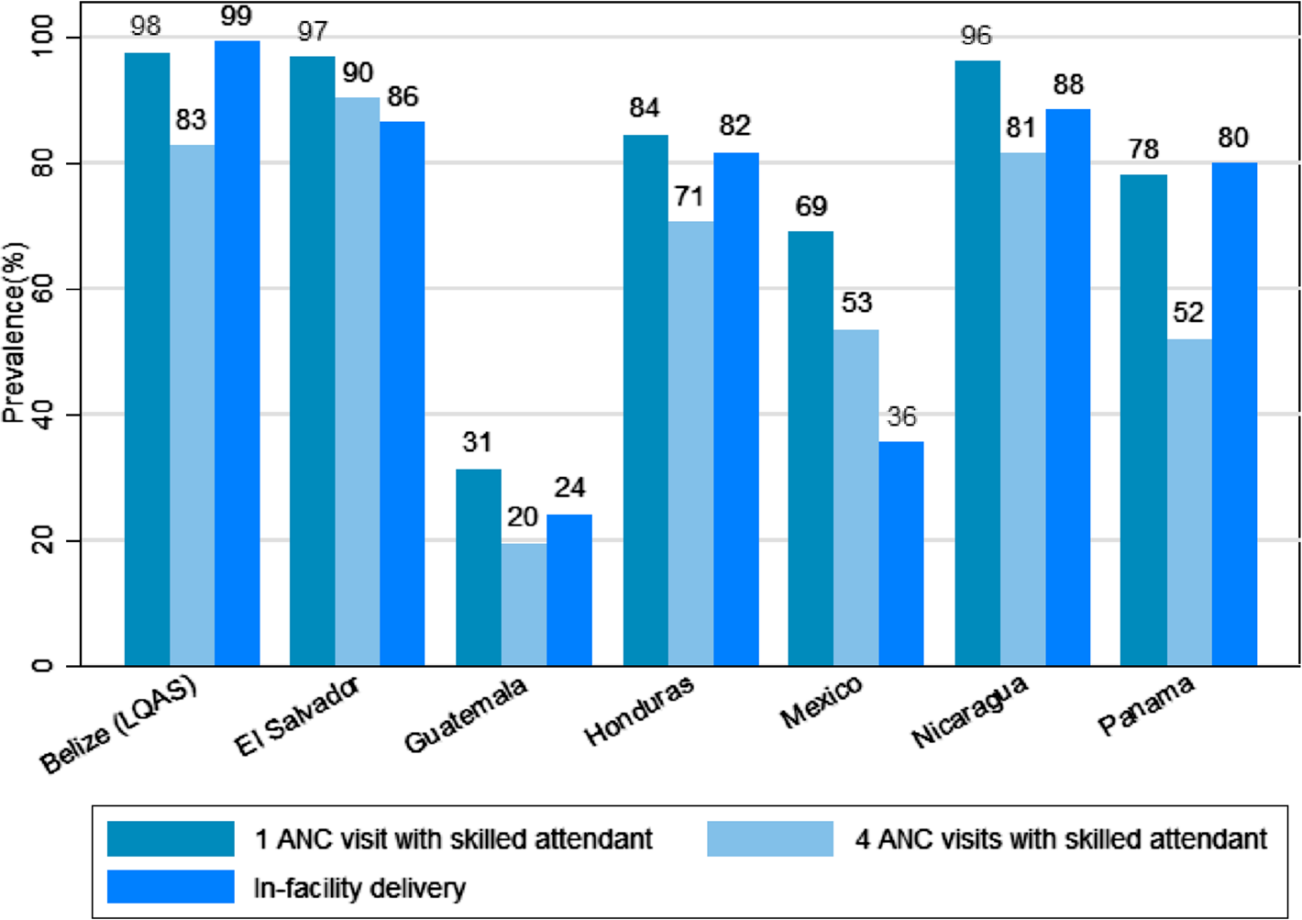
**Antenatal and delivery care indicators by country.**

Figure 3 shows the coverage of antenatal care and in-facility delivery by country. Belize had the highest proportion of institutional deliveries (99%), while Guatemala had the lowest (24%). The proportion of women with four antenatal care visits with a skilled attendant was highest in El Salvador (90%) and the lowest in Guatemala (20%).

**Figure 4:**
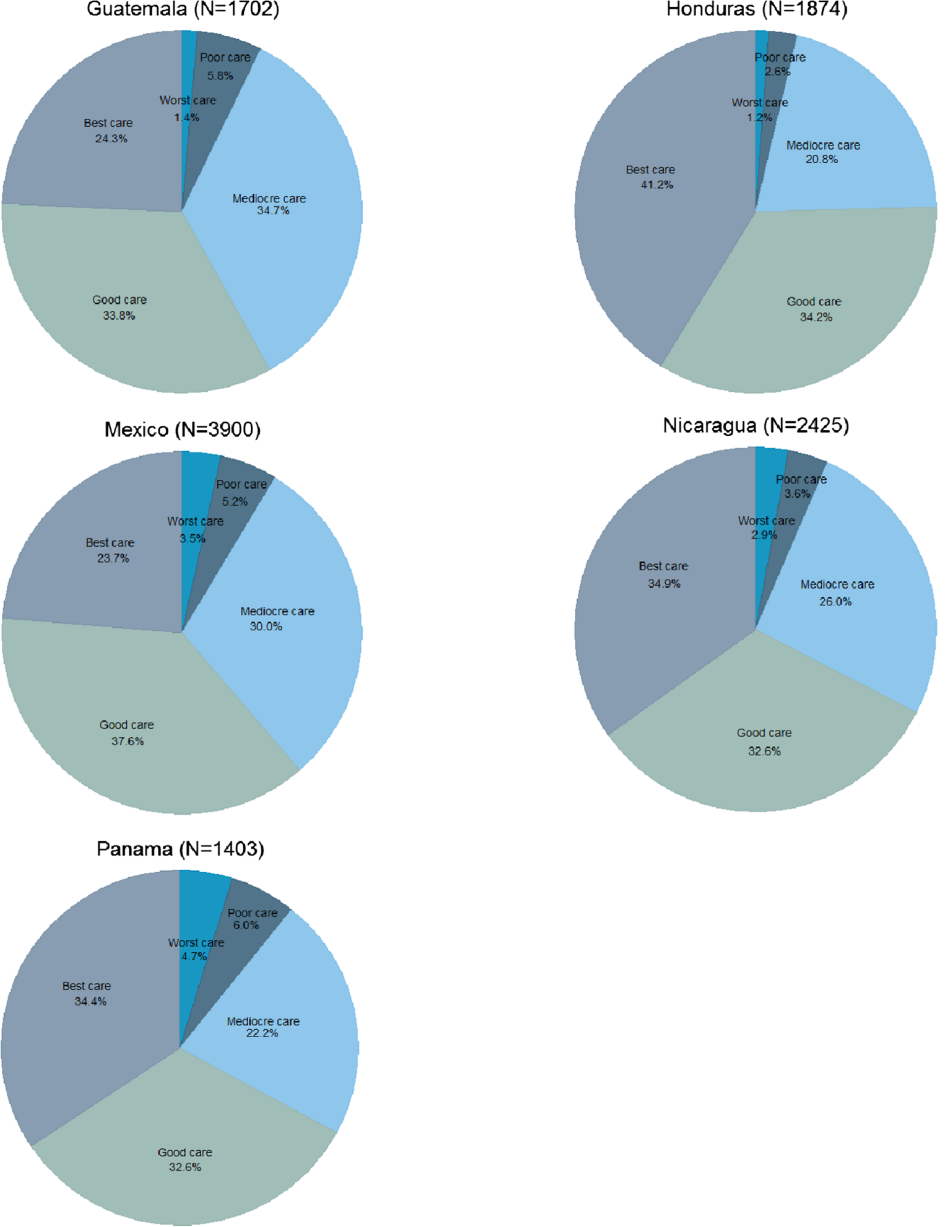
**Rating of overall quality of care for the most recent health facility visit, by country.**

A large percentage of surveyed women reported poor satisfaction with the quality of health services during their most recent visit to a health facility (Figure 4). The highest levels of satisfaction (those deeming the care “good” or “best”) were observed in Honduras (75%), while the lowest were observed in Guatemala (58%). The lack of interpreters or culturally sensitive materials and practices in these health facilities is likely to have impacted these high dissatisfaction rates.

Table 4 displays self-reported rates of sexual activity, condom use, and contraceptive use (oral, condom, intrauterine device (IUD), injectable, or withdrawal) in Costa Rica by grade. Girls were more likely than boys to be sexually active (defined as sexual contact within three months of survey). About 5% of students in 7th grade were sexually active, compared with 41% of students in grade 11. A large proportion of sexually active students reported using contraceptives (88%), but a lower percentage reported using condoms (69%).

**Table 4:** **Self-reported sexual activity and contraceptive among students in Costa Rica, by grade**

**Grade**	**Gender**	**Number of students**	**Sexually active**			Condom use, among sexually active students*			Contraceptive use, among sexually active students*		
			***N***	***%***	***SE (%)***	***N***	***%***	***SE (%)***	***N***	***%***	***SE (%)***
**7**	Both	236	22	5	2	15	73	11	21	98	2
	Female	126	16	6	3	11	81	9	15	97	3
	Male	110	6	3	2	4	53	27	6	100	0
**8**	Both	217	41	12	4	22	51	11	34	82	8
	Female	127	24	16	6	8	30	16	18	72	15
	Male	88	16	8	4	14	93	4	15	98	2
**9**	Both	214	43	27	7	31	69	16	38	96	2
	Female	140	28	28	10	17	57	21	23	94	4
	Male	72	14	26	9	14	100	-	14	100	0
**10**	Both	202	46	21	4	32	75	6	37	81	7
	Female	131	27	22	6	16	70	7	20	77	8
	Male	71	19	20	7	16	86	10	17	90	10
**11**	Both	42	10	41	8	8	83	15	8	83	15
	Female	22	3	9	7	3	100	-	3	100	-
	Male	20	7	71	18	5	80	18	5	80	18

*Effective sample size of sexually active students may be smaller due to exclusion of observations with missing data on contraceptive use.

Availability of contraceptives at health facilities and the prevalence of stock-outs in the previous three months are shown in Table 5. Among the facilities surveyed, Nicaragua had the lowest availability of condoms on the day of the survey (82.5%). The proportion of facilities with oral and injectable contraceptives was lowest in Panama (75% in both categories). El Salvador had the lowest proportion of facilities with IUD in stock on the day of the survey (21.5%). Among facilities that were well-stocked on the day of the survey, there was wide variation in reports of recent stock-outs by country and method of contraception. Facilities in Mexico were more likely to report a recent stock-out, and stocks of injectable contraceptives were more likely to have been stocked out, as compared to male condoms or oral contraceptives.

**Table 5:** **Availability and stock-out of contraceptives in health facilities that reported storing contraceptives**

**Country**	**# of facilities that reported routinely storing contraceptives**	Availability on day of survey* (%)				# of facilities evaluated for previous months’ stock**	Reported stock-outs in facilities with male condoms, oral contraceptive, injectable contraceptive available on day of survey** (%)		
		***Male condom***	***Oral contraceptive***	***Injectable***	***IUD***		***Male condom***	***Oral pill***	***Injectable***
Belize	19	89.5	100	78.9	36.8	15	0.0	6.7	33.3
El Salvador	65	90.8	87.7	90.8	21.5	***	***	***	***
Guatemala	90	88.9	78.9	97.8	24.4	61	1.6	9.8	9.8
Honduras	89	96.6	94.4	94.4	70.8	79	0.0	5.1	2.5****
Mexico	73	93.2	87.7	86.3	58.9	40	5.0	17.5	20.0
Nicaragua	63	82.5	85.7	98.4	76.9	46	4.3	4.3	13.0
Panama	24	100	75.0	75.0	37.5	9	0.0	0.0	22.2

*Among facilities that reported routinely storing contraceptives.

**Stock-out of contraceptives in the three months prior to the day of the survey. These questions were only asked at ambulatory EONC facilities with availability of male condoms, oral contraceptives, and injectable contraceptives on the day of the survey and basic and complete EONC facilities with availability of male condoms, oral contraceptives, injectable contraceptives, and IUD on the day of the survey. Stock-outs not captured for IUD.

***Stock-outs not captured in El Salvador.

****Due to missing data points, 75 facilities asked about previous months’ stock of injectables instead of 79 facilities.

Table 6 shows the availability of vaccines at health facilities by country and the prevalence of stock-outs in the previous three months. Belize had the greatest number of vaccines in stock on the day of the survey and no stock-outs in the previous three months. Facilities in Mexico reported the most stock-outs for the measles, mumps, and rubella vaccine (MMR) and Bacillus Calmette-Guerin vaccine (BCG) and the lowest availability of pneumonia and pentavalent vaccines.

**Table 6:** **Availability and stock-out of vaccines in health facilities that reported routinely storing vaccines**

**Country**	**# of facilities that reported routinely storing vaccines**	Availability on day of survey* (%)					# of facilities evaluated for previous months’ stock**	Stock-outs in facilities with all vaccines available on day of survey** (%)	
		***BCG***	***MMR***	***Pentavalent***	***Pneumonia***	***Rotavirus***		***BCG***	***MMR***
Belize	10	100	100	100	***	***	9	0.0	0.0
El Salvador	28	78.6	76.9	96.4	71.4	75.0	***	****	****
Guatemala	55	96.4	96.4	96.4	92.7	90.9	33	3.0	3.0
Honduras	84	86.9	77.4	78.6	76.2	75.0	9	22.2	11.1
Mexico	37	73.0	83.8	78.4	40.5	78.4	7	42.9	57.1
Nicaragua	41	31.7	95.1	97.6	65.9	92.7	1	0.0	0.0
Panama	14	100	92.9	100	100	100	11	9.1	9.1

*Among facilities that reported routinely storing vaccines.

**Stock-out of vaccines in the three months prior to the day of the survey, not including availability on the day of the survey. These questions were only asked of facilities with all vaccines in stock on the day of the survey.

***Pneumonia and Rotavirus vaccines not measured in Belize.

****Stock-outs not captured for El Salvador. Influenza and Polio vaccines were observed but not included in analysis due to the fact that these vaccines are only administered at certain times of the year and therefore are not always expected to be in stock at facilities that routinely store other vaccines.

There were substantial differences between estimates of key health indicators derived from our surveys, which targeted the most disadvantaged populations, and estimates derived from previous national surveys (Table 7). We restricted our age groups for this comparison to match previously available data. In Nicaragua and Mexico, our estimates were equal to or lower than previous national estimates, with the largest differences observed for skilled birth attendance and MMR immunization in Chiapas. In other countries, performance compared to national estimates varied widely. In Panama, unmet need for contraception was 61 percentage points higher than the national average. In El Salvador, timely initiation of breastfeeding was 34 percentage points higher than the national estimates. In Guatemala, skilled birth attendance was 32 percentage points lower than the national average. In Belize, our estimates were often higher than those of the 2006 Multiple Indicator Cluster Survey, but this difference may be attributable to the seven-year lag between the surveys or our reliance on a convenience sample for our survey.

**Table 7:** **Comparison of selected indicators to national estimates**

**Indicator**	**Country**	**SM2015 survey estimate**	National estimate*	**Difference**
1 skilled antenatal care visit, all births in the past 2 years	Belize	98%	94%	4%
Institutional delivery, all births in the past 2 years	Belize	99%	88%	11%
MMR immunization†	Belize	87%	82%	5%
Full immunization†	Belize	68%	56%	12%
Exclusive breastfeeding, children aged 0–5 months	Belize	33%	10%	23%
Oral rehydration therapy	Belize	73%	61%	13%
Unmet need for contraception	Belize	28%	31%	−4%
1 skilled antenatal care visit, all births in the past 5 years	Nicaragua	94%	95%	−1%
4 skilled antenatal care visits, all births in the past 5 years	Nicaragua	80%	93%	−14%
Institutional delivery, all births in the past 5 years	Nicaragua	87%	88%	−1%
Measles immunization for children aged 12–29 months†	Nicaragua	88%	88%	0%
1 or more antenatal care visits for the most recent pregnancy in the past 5 years, among women 20–49 years old	Mexico	94%	99%	−5%
Anemia, children aged 12–59 months	Mexico	25%	24%	0%
MMR immunization, children aged 12–23 months, according to health card only	Mexico	49%	81%	−32%
Wasting (<−2SD weight for height)	Mexico	1%	2%	0%
Unmet need for contraception	Panama	88%	27%	61%
1 skilled antenatal care visit, all births in the past 5 years	Panama	75%	96%	−21%
Institutional delivery, all births in the past 5 years	Panama	79%	88%	−10%
Exclusive breastfeeding, children 0–5 months	Panama	45%	28%	17%
Anemia, 6–59 months	Honduras	26%	29%	−4%
MMR coverage for children aged 12–23 months†	Honduras	94%	88%	6%
Contraceptive prevalence, modern methods	Honduras	70%	64%	6%
1 skilled antenatal care visit, for most recent birth in the past 5 years	Honduras	79%	97%	−18%
Breastfeeding initiated within an hour of birth, most recent birth in past 5 years	Honduras	74%	64%	10%
Contraceptive prevalence, any method	El Salvador	33%	73%	−40%
1 antenatal care visit, any attendant, all births in last 5 years	El Salvador	98%	94%	4%
Timely initiation of breastfeeding, all births in last 5 years	El Salvador	67%	33%	34%
Exclusive breastfeeding, children aged 0–5 months	El Salvador	60%	31%	29%
Stunting	El Salvador	16%	19%	−3%
Anemia, children aged 12–59 months	El Salvador	25%	23%	2%
Oral rehydration therapy	El Salvador	64%	51%	13%
Condom use at last sexual intercourse, women aged 15-19	Costa Rica	64%	44%	20%
Measles immunization**^†^	Guatemala	88%	76%	13%
DPT immunization**^†^	Guatemala	87%	83%	4%
1 antenatal care visit, any attendant, most recent birth in last 5 years	Guatemala	s80%	83%	−3%
Skilled birth attendance, most recent birth in last 5 years	Guatemala	23%	54%	−32%
Institutional delivery, most recent birth in last 5 years	Guatemala	23%	53%	−31%

*Sourcesnn Belize: 2006 Multiple Indicator Cluster Survey (MICS). Guatemala: 2006 Encuesta de condiciones de vida (ENCOVI). Honduras: 2011–2012 Demographic and Health Survey (DHS/ENDESA). Mexico: 2011–2012 National Health and Nutrition Survey (ENSANUT). Panama: 2009 National Survey of Sexual and Reproductive Health (ENASSER). El Salvador: 2008 Reproductive Health Survey (RHS/FESAL).

**National estimate for children under 6 years.

^†^Based on vaccine card and caregiver recall.

Child indicators include aged 0–59 months unless otherwise noted.

## Discussion

To our knowledge, this is the largest study conducted in poor areas of Mesoamerica. The comparable, cross-country nature of these surveys allows us to uncover large disparities between and within countries in terms of health behaviors, risk factors, and availability of medicines and services in health facilities. These findings provide the baseline for SM2015’s result-based interventions and enable the countries to effectively target services and geographic areas. Moreover, our surveys will allow us to link household health practices with availability of medicines and services at nearby health facilities to determine whether poor outcomes are due to facility or household factors in future analyses.

Our studies enabled us to better understand the health situation of the population under study and their needs. We focused on stock outs in our indicators for services rather than the amount of dispensed drugs or services provided in order to ensure that the facilities are able to properly function. The health facilities are designed to provide for a well-known population and should be stocked to adequately provide services to their target populations. Though it is possible that certain facilities may see a change in demand due to growth or a shift in health-seeking behaviors once a facility is known for good services and availability of drugs, in the long run, the demand should stabilize and the facilities and health authorities can be expected to maintain adequate supplies.

Comparisons between our indicator estimates and national estimates from previous surveys highlight the fact that national-level indicators mask large disparities in health service delivery and health outcomes within the population. It is also important to note that many previous national surveys lacked sufficient sample sizes to generate precise estimates for the poorest populations. The large sample sizes in SM2015 surveys allow us to better assess the experience of the underserved and the magnitude of disparities. Indeed, for some indicators, SM2015 areas showed a better performance than the national average. However, in general, the SM2015 estimates showed poor performance compared to the country as a whole.

Our findings revealed that there was availability of contraceptives at health facilities but a low uptake from the population. This could be due to the fact that SM2015 led to a rise in availability but not in demand. It is possible that the population is not receptive to the concept of using contraceptives due to religion and culture. On the other hand, it is possible that the population is not aware of the increase in supplies in this short period of time and that future surveys among the population will capture an increase in use. Whatever the reason for this finding, programs to educate women about the importance of birth spacing and the availability of contraceptive methods should be implemented. Moreover, these educational programs should include elders and other family members such as mothers or mothers-in-law.

The surprising finding that girls in 11th grade in Costa Rica were less likely to be sexually active may be because girls who defer their sexual activity to a later age are more likely to stay in school. Several studies in low- and middle-income countries have found that retention in school is associated with delayed sexual debut [25-30]. School retention and higher education levels are, in turn, associated with increased contraceptive use, delayed age of marriage, and reduced risk of adolescent pregnancy [25,28,31,32], a key outcome for SM2015 in Costa Rica. Further investigation is needed to understand the relationship between sexual activity and schooling in Costa Rica. It is also important to mention that our sample size is very small for this age group and thus the standard errors are sizeable for these point estimates.

Furthermore, the response rate in Costa Rica was very low. However, a low response rate does not necessarily imply that the results were biased [22,23]. We did not find any association between response rates and socio-economic status or crude death rates across the geographic regions we surveyed. Likewise, we did not find any association between response rate and sexual behavior indicators across regions. Still, it is possible that respondents differ from non-respondents for some survey outcomes. Unfortunately, our low response was due to the lack of consent from parents, rather than students declining to take the survey. The low response rate is similar to what has been observed before [22]. Furthermore, previous studies have reported that teens do not adequately report their sexual behaviors [33]. However, we hope that we will be able to capture a trend among the respondents even if they may not be representative of the population. Basically, we are comparing the same two groups that decided to take our surveys at baseline and follow-up. SM2015 should explore means to increase the response rate in the future by using incentives or other means.

It is disturbing that, across countries, 25% or more of our respondents were not satisfied with the quality of services provided at health facilities. Building and staffing health facilities in these poor, sometimes remote, regions is challenging for governments, and their efforts should be commended. However, if these populations are to be well-served, remedying dissatisfaction should be a key focus. Ensuring satisfaction with health services can stimulate health care-seeking behavior [34]. Indeed, there is a likely association between poor satisfaction and the availability of interpreters or culturally sensitive materials and practices in these health facilities. Thus, in these low-resource settings, ensuring health workers offer culturally sensitive care for their patients may be an easy way to encourage households to seek care.

Vaccine stock-outs are of particular concern in Mexico. Discussions with health authorities revealed that a shortage of the pneumonia vaccine occurred during the study period. Unfortunately, we do not have historic data at the level of the health facility for time periods prior to our study, except for government reports. Efforts by countries to ensure the availability of vaccines are likely to increase visits to clinics [35]. Indeed, if women bring their children to a facility and do not receive vaccines nor drugs, they may be less likely to return.

Surveying both contraceptive use in households and availability in health facilities allows us to look at both demand and supply. We found that health facilities generally had stocks of contraceptives, although supplies were lacking in certain facilities. In household surveys, use of contraceptives was the most controversial topic, and garnering responses sometimes posed a challenge for interviewers. Community elders resisted the inclusion of family planning questions in our surveys, but younger women generally answered readily. To address potential resistance, in every country we held meetings with communities to explain our objectives and discuss the content of the survey. Despite these efforts, our survey was temporarily halted by local leaders in Panama due to the contentious nature of these survey questions. At the same time, very few surveyed women refused to respond to family planning questions in any of the countries. There is a clear generational gap between old habits and beliefs and contemporary health behaviors, and thus, engaging older generations in the promotion of contraceptive use, in addition to women of reproductive age, may encourage more widespread utilization.

With respect to nutrition, large disparities within the poor regions of Mesoamerica were observed. A notable proportion of children surveyed were overweight or obese, while a smaller percentage was malnourished. This finding calls for more investigation into why successful malnutrition reduction has succeeded in certain communities but not others. This finding also highlights the need to address the chronic disease risk factors emerging among children and young adults in these populations.

Further research is required to understand why the prevalence of anemia was particularly high in Panama. It is crucial to ensure that infections are not causing these high rates. Iron supplementation and deworming campaigns could reduce these rates. Many of these areas were very remote and our interviewers had to use several modes of transportation to reach them. Hence, ensuring a steady supply of medicine and fortification in those areas requires multifaceted logistical planning and implementation.

Conducting our own census enabled us to better estimate total need for services in each area. Unfortunately, in many surveys, the focus is on estimating the correct numerator and relying on previous national censuses to enumerate the total number of conditions or behaviors. In our study there was large variation between our population counts and those provided by the central governments. For example, in Guatemala we identified 22,107 households, whereas the most recent national census (2002) identified only 18,491. Had we relied on national census data, we would have informed local health authorities of an inaccurate number of women and children in need of certain services. This finding calls for careful consideration when using previous censuses, even if they are relatively recent. The difference between the true denominator and that of a former census may be larger in poor areas where the population is more likely to move around to seek employment or services.

SM2015 is an ambitious program to improve health in poor areas throughout Mesoamerica. Our study highlights the breadth and depth of the challenges involved, including wide-ranging disparities in SM2015 areas. Addressing these issues and meeting the targets set by the Initiative will be no easy task. However, our ability to document these issues at an early stage in the implementation of SM2015 is a great step toward these targets. Our study has provided a reliable baseline of data from which the Initiative can build its activities. The ongoing assessment furnished by the innovative design of SM2015 has already led to increased focus on local challenges and the fine-tuning of intervention approaches. The Initiative’s monitoring and evaluation framework will allow health officials with limited resources to identify and target areas of greatest need and verify the results of the efforts. These data provide a baseline of much-needed information for evidence-based action on health throughout Mesoamerica.

## Additional file

Additional file 1: Table S1. Maternal and child health indicators by country.

## Abbreviations

CAPI: Computer‐assisted personal interview
DBS: Dried blood spot
EONC: Essential obstetric and neonatal care
IDB: Inter-American Development Bank
IRB: Institutional review board
MRR: Medical record review
PSU: Primary sampling unit
SM2015: Salud Mesoamérica 2015
